# Prevalence and risk factors for significant liver fibrosis among HIV-monoinfected patients

**DOI:** 10.1186/1471-2334-10-116

**Published:** 2010-05-13

**Authors:** Michelle DallaPiazza, Valerianna K Amorosa, Russell Localio, Jay R Kostman, Vincent Lo Re

**Affiliations:** 1 Division of Infectious Diseases, Department of Medicine, New York University School of Medicine, New York, NY, USA; 2 Division of Infectious Diseases, Department of Medicine, University of Pennsylvania School of Medicine, Philadelphia, PA, USA; 3 Infectious Diseases Section, Philadelphia Veterans Affairs Medical Center, Philadelphia, PA, USA; 4 Department of Biostatistics and Epidemiology and Center for Clinical Epidemiology and Biostatistics, University of Pennsylvania School of Medicine, Philadelphia, PA, USA

## Abstract

**Background:**

HIV-monoinfected patients may be at risk for significant liver fibrosis, but its prevalence and determinants in these patients are unknown. Since HIV-monoinfected patients do not routinely undergo liver biopsy, we evaluated the prevalence and risk factors of significant hepatic fibrosis in this group using the aspartate aminotransferase (AST)-to-platelet ratio index (APRI).

**Methods:**

We conducted a cross-sectional study among HIV-infected patients negative for hepatitis B surface antigen and hepatitis C antibody in the Penn Center for AIDS Research Adult/Adolescent Database. Clinical and laboratory data were collected from the database at enrollment. Hypothesized determinants of significant fibrosis were modifiable risk factors associated with liver disease progression, hepatic fibrosis, or hepatotoxicity, including immune dysfunction (i.e., CD4 T lymphocyte count <200 cells/mm^3^, HIV viremia), diseases associated with hepatic steatosis (e.g., obesity, diabetes mellitus), and use of antiretroviral therapy. The primary outcome was an APRI score >1.5, which suggests significant hepatic fibrosis. Multivariable logistic regression identified independent risk factors for significant fibrosis by APRI.

**Results:**

Among 432 HIV-monoinfected patients enrolled in the CFAR Database between November 1999 and May 2008, significant fibrosis by APRI was identified in 36 (8.3%; 95% CI, 5.9 - 11.4%) patients. After controlling for all other hypothesized risk factors as well as active alcohol use and site, detectable HIV viremia (adjusted OR, 2.56; 95% CI, 1.02 - 8.87) and diabetes mellitus (adjusted OR, 3.15; 95% CI, 1.12 - 10.10) remained associated with significant fibrosis by APRI.

**Conclusions:**

Significant fibrosis by APRI score was found in 8% of HIV-monoinfected patients. Detectable HIV viremia and diabetes mellitus were associated with significant fibrosis. Future studies should explore mechanisms for fibrosis in HIV-monoinfected patients.

## Background

As survival of HIV-infected individuals has improved with widespread use of combination antiretroviral therapy (ART), non-HIV-related conditions are now common causes of morbidity among HIV-infected patients in the developed world. In particular, liver disease has emerged as an increasingly significant contributor to mortality among HIV-infected patients due to the high prevalence of viral hepatitis coinfection [1]. However, additional factors apart from viral hepatitis could contribute to hepatic fibrosis, including antiretroviral medications [2,3], concomitant metabolic diseases [4], and immunosuppression [5]. Consequently, HIV-infected patients without viral hepatitis coinfection might also be at risk for liver disease. However, few studies have examined the prevalence and potential risk factors for significant hepatic fibrosis among HIV-monoinfected patients (i.e., those without viral hepatitis coinfection) [2,6,7]. Identifying risk factors, particularly those that are modifiable, could help reduce the risk of liver disease in this population, especially as it ages.

To address this issue, we evaluated the prevalence and risk factors for significant hepatic fibrosis among HIV-monoinfected patients. Since liver biopsy results are generally not available on sufficiently large numbers of HIV-monoinfected patients to permit appropriate epidemiologic analyses, we used a non-invasive measure of significant liver fibrosis, the aspartate aminotransferase (AST)-to-platelet ratio index (APRI). This index has previously been validated as a surrogate marker of significant hepatic fibrosis in HIV/HCV-coinfected patients [8-10], and has recently been used to determine advanced fibrosis in HIV-monoinfected patients [2]. Our hypothesized determinants of significant fibrosis were modifiable risk factors associated with liver disease progression, hepatic fibrosis, or hepatotoxicity, including immune dysfunction (i.e., CD4 T lymphocyte count <200 cells/mm^3^, HIV viremia) [11], diseases associated with hepatic steatosis (e.g., obesity, diabetes mellitus) [12-14], and ART use [15,16].

## Methods

### Study Design and Patients

We conducted a cross-sectional study of patients enrolled in the Penn Center for AIDS Research (CFAR) Adult/Adolescent Database. This patient registry was initiated in November 1999 to track demographic, clinical, and laboratory data from HIV-infected patients cared for at four University of Pennsylvania-affiliated adult-care hospitals (i.e., Hospital of the University of Pennsylvania [HUP], Penn Presbyterian Medical Center [PPMC], Philadelphia Veterans Affairs Medical Center, and Pennsylvania Hospital) and at the adolescent HIV clinic at the Children's Hospital of Philadelphia [17]. Subjects enrolled from the HUP, PPMC, and Pennsylvania Hospital sites are comparable with regards to age, sex, race, and ethnicity, but given different eligibility rules, the adolescent HIV clinic at the Children's Hospital of Philadelphia provides care to younger patients while the Philadelphia Veterans Affairs Medical Center cares for a primarily male population. Subjects in the Penn CFAR Database have laboratory-confirmed HIV infection, provide informed consent, and complete a standardized questionnaire that collects demographic, medical, psychosocial, and HIV data. Additional laboratory data, such as results of hepatitis B surface antigen (HBsAg; Elecsys 2010; Roche Diagnostics, Indianapolis, IN), hepatitis C virus (HCV) antibody (anti-HCV; Abbott HCV EIA 2.0 or 3.0 enzyme immunoassay; Abbott Laboratories, Abbott Park, IL), liver aminotransferases, and platelet counts, can be downloaded into the Penn CFAR Database from hospital laboratory computer systems at the HUP and PPMC sites. The study was approved by the Institutional Review Board of the University of Pennsylvania.

All HBsAg-negative/anti-HCV-negative patients enrolled in the CFAR database at the HUP and PPMC sites between November 1, 1999 and May 31, 2008 were eligible for inclusion. A total of 400 patients was targeted to have 80% power to detect a 20% difference in the prevalence of significant liver fibrosis by APRI score between those with and without each risk factor of interest.

### Study Outcome

The main study outcome was significant fibrosis as determined by APRI score, a non-invasive measure of significant liver fibrosis. The APRI score is defined as: (AST[/ULN]*100/platelet count[10^9^/L]) [18]. APRI accurately represents liver fibrosis only when liver disease has reached a significantly severe stage. An APRI score >1.5 has been shown to be predictive of significant liver fibrosis (defined as an Ishak fibrosis score >3) with an area under the receiver operating characteristic curve of 0.76-0.85 [9,10,18]. Thus, we used the established cutoff of 1.5 to determine the presence of significant fibrosis.

### Data Collection

All data were collected from the Penn CFAR Database at subjects' enrollment and included: date of birth; sex; race/ethnicity; duration of HIV diagnosis; risk factors for HIV; self-reported alcohol use within the past 30 days (yes/no); self-reported marijuana use in the last 30 days (yes/no); diabetes mellitus (based on a random glucose level greater than 200 mg/dL and/or self-reported use of an anti-diabetic medication); obesity (defined as body mass index (BMI) ≥ 30 kg/m^2^); use of ART (defined as receipt of three antiretroviral medications from at least two different drug classes) [19]; CD4 cell count; HIV viral load (determined by Versant HIV-1 RNA 3.0 Assay; Bayer Diagnostics; lower limit of detection: 75 copies/mL); AST; and platelet count.

### Statistical analysis

BMI was calculated as body weight (kg)/height^2 ^(m^2^). Differences between patients with and without significant fibrosis by APRI score were assessed using Chi-square or Fisher's exact tests, as indicated, for categorical data and Wilcoxon rank-sum tests for continuous data.

Multivariable logistic regression was used to determine risk factors for significant fibrosis by APRI score. Hypothesized risk factors for significant fibrosis included a CD4 cell count below 200 cells/mm^3^, detectable HIV viremia (HIV viral load >75 copies/mL), diabetes mellitus, obesity, and ART use. Potential confounders evaluated included age; sex; race/ethnicity; CFAR site; duration of HIV; HIV risk factors; alcohol use; and marijuana use. Further model reduction was achieved by eliminating factors that proved to be potential risk factors only infrequently in 1000 bootstrap samples of the data [20]. Robust 95% confidence intervals (CIs) that do not depend on large-sample assumptions were then estimated using bias-corrected (accelerated) bounds from another round of 999 bootstrap re-samples [20]. All data were analyzed using Stata 10.1 (Stata Corp, College Station, TX).

## Results

Among 1,533 HIV-infected patients enrolled in the CFAR Database through May 31, 2008, 590 (38%) were from the HUP and PPMC sites. Among these patients, 50 (8%) were HBsAg-positive, 106 (18%) were anti-HCV-positive, 2 (0.3%) were HBsAg-positive and anti-HCV-positive, and 432 (73%) were HIV-monoinfected. The baseline characteristics of the 432 HIV-monoinfected subjects are shown in Table 1. Consistent with the population served by the affiliated hospitals, subjects in this study were primarily male (59%) and African American (72%). The majority (63%) received ART, and the median duration of HIV diagnosis was 2 years (interquartile range [IQR], 1 - 7 years). The median CD4 cell count was 340 cells/mm^3 ^(IQR, 177 - 516 cells/mm^3^), and the median HIV viral load was 4,950 copies/mL (IQR, 75 - 44,162 copies/mL).

**Table 1:** Baseline subject characteristics, overall and by significant fibrosis as determined by AST-to-platelet ratio index (APRI) score (APRI >1.5).

Characteristic	All Subjects (n = 432)	APRI ≤ 1.5 (n = 396)	APRI >1.5 (n = 36)	P-Value
**Median age (yrs, IQR)**	38 (32-44)	38 (31-44)	39 (35-45)	0.4

**Male sex (no., %)**	254 (59%)	228 (58%)	26 (72%)	0.09

**Race (no., %)**				
**African-American**	311 (72%)	286 (72%)	25 (69%)	0.7
**Caucasian**	121 (28%)	110 (28%)	11 (31%)	

**Hispanic (no., %)**	17 (4%)	16 (4%)	1 (3%)	0.7*

**Alcohol use in last 30 days (no., %)**	185 (43%)	167 (42%)	18 (50%)	0.4

**Marijuana use in last 30 days (no., %)**	81 (19%)	72 (18%)	9 (25%)	0.3

**Diabetes mellitus (no., %)**	34 (8%)	28 (7%)	6 (17%)	0.04

**Body mass index (kg/m^2^, IQR)**				0.1*
Underweight (≤ 18.4 kg/m^2^)	15 (3%)	14 (4%)	1 (3%)	
Ideal (18.5 kg/m^2 ^- 24.9 kg/m^2^)	202 (47%)	179 (45%)	23 (64%)	
Overweight (25.0 kg/m^2 ^- 29.9 kg/m^2^)	135 (31%)	128 (32%)	7 (19%)	
Obese (30 kg/m^2 ^- 39.9 kg/m^2^)	65 (15%)	60 (15%)	5 (14%)	
Very Obese (≥ 40.0 kg/m^2^)	15 (3%)	15 (4%)	0	

**Median duration of HIV (yrs, IQR)**	2 (1-7)	2 (0-7)	2 (0-9.5)	0.8

**On antiretroviral therapy (no., %)**	273 (63%)	252 (63%)	21 (58%)	0.5

**Lopinavir/ritonavir (no., %)**				
**Prior**	175 (41%)	159 (40%)	16 (44%)	0.6
**Current**	74 (17%)	68 (17%)	6 (17%)	0.9

**Nevirapine (no., %)**				
**Prior**	67 (16%)	61 (15%)	6 (17%)	0.8
**Current**	12 (3%)	12 (3%)	0 (0%)	0.3*

**Didanosine (no., %)**				
**Prior**	57 (13%)	55 (14%)	2 (6%)	0.2*
**Current**	11 (3%)	11 (3%)	0 (0%)	0.3*

**Stavudine (no., %)**				
**Prior**	84 (19%)	78 (20%)	6 (17%)	0.7
**Current**	11 (3%)	5 (2%)	0 (0%)	0.5*

**Zidovudine/lamivudine/abacavir (no., %)**				
**Prior**	35 (8%)	33 (8%)	2 (6%)	0.6
**Current**	8 (2%)	8 (2%)	0 (0%)	0.4

**Median CD4 count (cells/mm^3^, IQR)**	340 (177-516)	342 (182-516)	315 (58-534)	0.3

**CD4 count <200 cells/mm^3 ^(no., %)**	121 (28%)	107 (27%)	14 (39%)	0.1

**Median HIV viral load (copies/mL, IQR)**	4,950 (75-44,162)	2,506 (75-33,228)	38,517 (2,958-206,373)	0.0004

**HIV viral load category (no., %)**				
**75-49,999 copies/mL**	331 (76%)	311 (78%)	20 (56%)	0.003*
**50,000-99,999 copies/mL**	33 (8%)	30 (8%)	3 (8%)	
**≥ 100,000 copies/mL**	68 (16%)	55 (14%)	13 (36%)	

**Detectable HIV viremia (>75 copies/mL)**	323 (75%)	291 (73%)	32 (89%)	0.04

**Median aspartate aminotransferase (U/L, IQR)**	39 (23-42)	29 (23-37)	84 (65-123)	<0.001

**Median platelet count (×10^9^/L, IQR)**	232 (187-283)	237 (197-287)	130 (92-163)	<0.001

**Median APRI**	0.42 (0.31-0.66)	0.40 (0.30-0.59)	2.1 (1.7-80-3.64)	<0.001

* P-values for differences between subjects determined by Fisher's exact test

IQR = interquartile range

Thirty-six patients (8.3%; 95% CI, 5.9 - 11.4%) had significant liver fibrosis as determined by APRI score. Age; sex; race; ethnicity; BMI; duration of HIV diagnosis; use of ART, alcohol, and marijuana; and median CD4 cell count were similar between subjects with and without significant fibrosis by APRI score (Table 1). Patients with significant fibrosis were more likely to have diabetes mellitus (6 [17%] versus 28 [7%]; p = 0.04) and detectable HIV viremia (32 [89%] versus 291 [73%]; p = 0.0004).

Table 2 shows results of the multivariable analysis with bootstrap re-sampling examining risk factors for significant fibrosis by APRI. After controlling for all other hypothesized risk factors as well as active alcohol use and site, detectable HIV viremia (adjusted OR, 2.56; 95% CI, 1.02 - 8.87) and diabetes mellitus (adjusted OR, 3.15; 95% CI, 1.12 - 10.10) remained associated with significant fibrosis by APRI score. CD4 count below 200 cells/mm^3^, obesity, and ART use did not increase the risk of significant fibrosis. Additionally, when we examined CD4 cell count as a continuous variable, the OR of significant fibrosis did not decrease with each 50 cells/mm^3 ^increase in CD4 cell count (adjusted OR, 0.98; 95% CI, 0.90 - 1.06).

**Table 2:** Evaluation of risk factors for significant fibrosis by AST-to-platelet ratio index score (APRI>1.5) among HIV-monoinfected patients.

Risk Factor	Odds Ratio (95% Confidence Interval)*
**Diabetes mellitus**	3.15 (1.12, 10.10)

**Obesity**	0.70 (0.22, 2.24)

**Detectable HIV viremia**^‡^	2.56 (1.02, 8.87)

**CD4 count <200 cells/mm^3^**	1.48 (0.69, 3.17)

**Antiretroviral therapy use**	0.90 (0.39, 2.07)

^‡ ^Defined as HIV RNA > 75 copies/mL

* Odds ratios obtained via bootstrap re-sampling with bias-corrected (accelerated) confidence intervals. Each risk factor was adjusted for the other risk factors as well as alcohol use and site.

## Discussion

In this study, we found the prevalence of significant liver fibrosis as determined by APRI score to be 8.3% among HIV-monoinfected patients. In addition, both diabetes mellitus and HIV viremia were risk factors for significant fibrosis by APRI in this sample. Other hypothesized risk factors, including CD4 count below 200 cells/mm^3^, obesity, and ART use were not associated with significant fibrosis by APRI score.

The prevalence of significant liver fibrosis among HIV-monoinfected patients in this study sample is consistent with a prior cross-sectional study conducted among 1,845 HIV-infected patients without viral hepatitis at the Johns Hopkins HIV Clinic, which showed that 7% of these patients had significant hepatic fibrosis as determined by APRI >1.5 [7]. Predictors of significant fibrosis included CD4 cell count ≥ 200 cells/mm^3 ^(adjusted OR, 0.29; 95% CI, 0.12 - 0.69), random glucose ≥ 140 mg/dL (adjusted OR, 2.76; 95% CI, 1.06 - 7.16), and active ART use (adjusted OR, 2.93; 95% CI, 1.22 - 7.03). In contrast, a cross-sectional analysis among 1,307 HIV-monoinfected patients receiving care at an HIV clinic in Spain showed that only 1% had significant liver fibrosis as measured by transient elastometry [6]. Differences in the non-invasive methods to measure hepatic fibrosis and in the study populations might account for the disparate results.

Our study found that diabetes mellitus was a risk factor for significant liver fibrosis among HIV-monoinfected patients. The biological mechanism underlying the association between diabetes and liver fibrosis remains poorly understood. Diabetes can promote hepatic steatosis [21], which can lead to hepatic fibrosis, but additional evidence suggests that diabetes can induce liver fibrosis independent of steatosis and visceral obesity [14]. This mechanism may be related to the ability of insulin to stimulate hepatic stellate cells [22]. Among HIV-uninfected patients, diabetes mellitus has been associated with advanced liver fibrosis in patients with chronic HCV infection [23-25]. Further, several studies have identified diabetes as determinant of advanced fibrosis [26,27] and increased fibrosis progression [28] among HIV-uninfected patients with nonalcoholic fatty liver disease. We were unable to explore the impact of severity of diabetes (e.g., hemoglobin A1c) and anti-diabetic medications on significant fibrosis since the Penn CFAR Adult/Adolescent Database does not collect data on these variables. Additional research is needed to examine how diabetes and its treatment might contribute to hepatic fibrosis in HIV and to evaluate the effect of glycemic control on liver fibrosis progression in HIV.

Another risk factor for significant hepatic fibrosis among HIV-monoinfected patients in this sample was HIV viremia. The influence of HIV viremia on hepatic fibrosis in HIV-monoinfected individuals is unclear. However, among HIV/HCV-coinfected patients, HIV accelerates the progression of hepatic fibrosis. In addition, a shorter time with an undetectable HIV viral load has been shown to increase the risk of hepatic decompensation among HIV/HCV-coinfected patients [11]. Results from the Strategies for Management of Antiretroviral Therapy (SMART) study showed that rates of hepatic disease were significantly higher among HIV-infected patients who interrupted ART compared with those who maintained it [29]. Our results are consistent with findings from *in vitro *studies in which HIV has been shown to enter and replicate in hepatic stellate cells and directly promote hepatic fibrosis [30]. Future studies should further evaluate this and other potential mechanisms by which HIV increases the risk of liver fibrosis.

Importantly, ART use was not associated with significant fibrosis. In this cross-sectional study, we were not able to assess the duration of exposure to ART. However, our finding is consistent with a recent report demonstrating that ART has a negligible effect on liver fibrosis progression as measured by APRI in HIV-monoinfected patients but is associated with an increase in fibrosis progression in HIV/HCV-coinfected individuals [2]. These results suggest that while antiretrovirals may cause hepatotoxicity, their impact on fibrosis in HIV-monoinfected patients may be limited. It is possible that older antiretroviral medications might have increased the risk of advanced liver fibrosis, but that this effect could be counterbalanced by a beneficial impact of newer regimens. We did compare use of selected older non-nucleoside (i.e., nevirapine) and nucleoside (i.e., didanosine, stavudine, and zidovudine) reverse transcriptase inhibitors between patients with and without significant hepatic fibrosis and found no differences in their use between the groups (Table 1). Further research is needed into the long-term effects of ART, and specific antiretroviral drugs, on the liver in HIV monoinfection.

Our study had several limitations. First, since cross-sectional studies evaluate exposure and disease status at the same time, this study design is limited in its ability to determine whether exposure preceded or resulted from disease. However, since significant hepatic fibrosis is not known to lead to the hypothesized risk factors of interest, the cross-sectional design remains appropriate. This design did limit our ability to evaluate change in variables of interest over time.

Second, we identified only 36 subjects with significant fibrosis, and this small number limits our statistical power for identifying risk factors for this outcome. However, we used two stages of bootstrap re-sampling to evaluate predictors of interest and obtain robust estimates of the association between these factors and significant fibrosis.

Third, multivariable analyses may not entirely eliminate residual confounding from unmeasured factors. In particular, duration of comorbid illnesses and cumulative exposure to hepatotoxic medications might be associated with hepatic fibrosis. The CFAR Database does not collect frequency or quantity of alcohol use, duration of comorbidities (e.g., diabetes mellitus), duration of ART use, or use of non-antiretroviral medications that might be hepatotoxic (e.g., acetaminophen, metformin). We therefore could not examine the relationship between these variables and significant fibrosis.

Fourth, patients in our sample were anti-HCV-negative, but HCV viremia may be present in the absence of detectable HCV antibodies in HIV, although this occurs only rarely [31,32]. The retrospective study design did not permit us to test patients' sera for HCV RNA.

Fifth, we cannot exclude the possibility that some individuals were misclassified by APRI due to conditions which can decrease the platelet count (e.g., HIV-associated idiopathic thrombocytopenic purpura) or which transiently increase serum AST levels (e.g., ART-associated hepatotoxicity). Hepatic fibrosis would be ideally assessed by liver biopsy, but the acceptability, cost, and risk of liver biopsy among HIV-monoinfected patients make such a study impractical. APRI has been validated to identify significant hepatic fibrosis in HIV/HCV-coinfected patients [8] with a high degree of accuracy, but it has not been validated against liver biopsy in HIV-monoinfected patients. APRI compares favorably with other non-invasive biomarkers such as the FIB-4, MULTIVIRC equation, and Forns index that do not incorporate the same parameters [33].

Finally, our exclusion of subjects from certain CFAR sites, particularly the Philadelphia Veterans Affairs Medical Center and adolescent HIV clinic at the Children's Hospital of Philadelphia, due to the inability to obtain computerized laboratory results, potentially limits the generalizability of the study results.

## Conclusion

Our study found that 8% of HIV-monoinfected patients had significant liver fibrosis by APRI. Diabetes mellitus and HIV viremia were both risk factors for significant fibrosis by APRI. These results suggest the need for future longitudinal studies to examine liver fibrosis in HIV-monoinfected patients.

## Competing interests

The authors declare that they have no competing interests.

## Authors' contributions

M.D., V.K.A., J.R.K. and V.L.R. participated in the conception and design of the study. M.D., A.R.L., and V.L.R. participated in data cleaning, analysis, and interpretations of results. M.D. and V.L.R collaborated on initial drafts of the manuscript. All other authors contributed further to manuscript edits, reviewed, and approved the final manuscript.

## Pre-publication history

The pre-publication history for this paper can be accessed here:

http://www.biomedcentral.com/1471-2334/10/116/prepub
